# Gestational Exposure to Cigarette Smoke Suppresses the Gasotransmitter H_2_S Biogenesis and the Effects Are Transmitted Transgenerationally

**DOI:** 10.3389/fimmu.2020.01628

**Published:** 2020-07-28

**Authors:** Shashi P. Singh, Dinesh Devadoss, Marko Manevski, Aryaz Sheybani, Teodora Ivanciuc, Vernat Exil, Hemant Agarwal, Veena Raizada, Roberto P. Garofalo, Hitendra S. Chand, Mohan L. Sopori

**Affiliations:** ^1^Respiratory Immunology Division, Lovelace Respiratory Research Institute, Albuquerque, NM, United States; ^2^Department of Immunology and Nanomedicine, Herbert Wertheim College of Medicine, Florida International University, Miami, FL, United States; ^3^Department of Microbiology and Immunology, Galveston, TX, United States; ^4^Department of Pediatrics, University of New Mexico Health Sciences Center, Albuquerque, NM, United States

**Keywords:** gestational cigarette smoke, H_2_S biogenesis, human placenta, lungs, transgenerational effect

## Abstract

**Rationale:** Gestational cigarette smoke (CS) impairs lung angiogenesis and alveolarization, promoting transgenerational development of asthma and bronchopulmonary dysplasia (BPD). Hydrogen sulfide (H_2_S), a proangiogenic, pro-alveolarization, and anti-asthmatic gasotransmitter is synthesized by cystathionine-γ-lyase (CSE), cystathionine-β-synthase (CBS), and *3-*mercaptopyruvate sulfur transferase (3MST).

**Objective:** Determine if gestational CS exposure affected the expression of H_2_S synthesizing enzymes in the mouse lung and human placenta.

**Methods:** Mice were exposed throughout gestational period to secondhand CS (SS) at approximating the dose of CS received by a pregnant woman sitting in a smoking bar for 3 h/days during pregnancy. Lungs from 7-days old control and SS-exposed pups and human placenta from mothers who were either non-smokers or smokers during pregnancy were analyzed for expression of the enzymes.

**Measurements:** Mouse lungs and human placentas were examined for the expression of CSE, CBS, and 3MST by immunohistochemical staining, qRT-PCR and/or Western blot (WB) analyses.

**Results:** Compared to controls, mouse lung exposed gestationally to SS had significantly lower levels of CSE, CBS, and 3MST. Moreover, the SS-induced suppression of CSE and CBS in F1 lungs was transmitted to the F2 generation without significant change in the magnitude of the suppression. These changes were associated with impaired epithelial-mesenchymal transition (EMT)—a process required for normal lung angiogenesis and alveolarization. Additionally, the placentas from mothers who smoked during pregnancy, expressed significantly lower levels of CSE, CBS, and 3MST, and the effects were partially moderated by quitting smoking during the first trimester.

**Conclusions:** Lung H_2_S synthesizing enzymes are downregulated by gestational CS and the effects are transmitted to F2 progeny. Smoking during pregnancy decreases H_2_S synthesizing enzymes is human placentas, which may correlate with the increased risk of asthma/BPD in children.

## Introduction

Maternal smoking during pregnancy remains relatively common (1, 2) and about 1/4th of mothers, who smoke during pregnancy, misreport as quitters (3). Epidemiological data and animal studies suggest that exposure to CS, including secondhand CS (SS) during pregnancy increases the risk of allergic asthma (AA) and BPD in the progeny (4–7); the latter encompasses alveolar simplification (8). Gestational exposure of mice to CS/SS impairs angiogenesis, exacerbates AA, and induces BPD-like alveolar simplification through downregulation of HIF-1α; this phenotype is transmitted to the F2 progeny (9–11). The mechanisms by which gestation CS promotes AA and BPD are unclear. H_2_S is the newest member of gasotransmitter that affects many physiological systems (12). H_2_S is an anti-inflammatory that promotes angiogenesis/vascularization and wound healing (7, 13). In the lung, H_2_S attenuates lipopolysaccharide-induced acute lung injury (14), confers protection against ventilation-induced pulmonary inflammation and injury (15), promotes alveolarization and airway development (16), and protects against asthma and allergic inflammation (17, 18).

In mammals, H_2_S is mainly produced from L-cysteine by three enzymes: cystathionine γ-lyase (CSE), cystathionine β-synthase (CBS), and 3-mercaptopyruvate sulfur transferase (3MST) (19, 20). The distribution of these enzymes in various tissue is somewhat uncertain. It is generally believed that CSE and CBS are the two most prominent H_2_S synthesizing enzymes, where CBS is primarily localized to the brain and CSE in non-neuronal tissues (21, 22). However, this is not an inflexible rule. For example, adult rat lung expresses CSE and 3MST, but insignificant levels of CBS (23), but CBS has been reported in airway vasculature and lung epithelial cells, and CSE is present in the lung parenchyma (16). All three H_2_S synthesizing enzymes (CSE, CBS, and 3MST) are present in the lungs of cows and sea lions (24) and lung biopsies from non-small cell lung cancer patients (25) and the lung epithelial cell line A549 (26) also express all the three enzymes. In a recent report, 3MST was shown to be upregulated in the lung adenocarcinoma (27). Similarly, while the portal vein and thoracic aorta contain CSE, ileum expresses both CSE and CBS (28). Thus, the expression of H_2_S enzymes depends on the tissue type and the state of cell differentiation.

Epithelial mesenchymal transition (EMT) is an important process for cell differentiation during development, organogenesis, and carcinogenesis (29, 30). While dysregulated EMT in the adult lung promotes multiple respiratory diseases, it is indispensable for the development of lung epithelium (31), where the TGF-β/Smad pathway plays a key role (31, 32). Although, H_2_S has been shown to inhibit EMT in lung cancers through Wnt/Catenin signaling and the activation of HIF-1α (25, 33), HIF-1α is dramatically downregulated by gestational CS in the 7-days old mouse lung (11, 34) and, in some lung injuries, H_2_S promotes EMT and lung repair (35, 36). Moreover, HIF-1α mediates cellular differentiation through TGF-β (37, 38)—a key participant in EMT (39, 40). Thus, EMT is important in lung development and organogenesis, and requires H2S-induced HIF-1α/TGF-β.

In this communication we demonstrate that gestational SS suppresses TGF-β, EMT, and anti-asthmatic factors, and the 7-days old mouse lung and human placentas contains all the three H_2_S synthesizing enzymes. Gestational exposure to CS suppresses the expression of these enzymes in the mouse lung and human placentas from mothers' who smoke during pregnancy. The latter prompts the possibility that the placental levels of H_2_S synthesizing enzymes may correlate with the risk of AA and BPD in children.

## Materials and Methods

### Animals

Pathogen-free BALB/c mice were purchased from the FCR Facility (Frederick, MD). The animals were housed at the Animal Facility of Lovelace Respiratory Research Institute, Albuquerque, NM in accordance with the Guidelines from the Association for the Assessment and Accreditation for Laboratory Animal Care International. Animals were kept in exposure chambers maintained at 26 ± 2°C with 12-h light/dark cycle. Food and water were provided *ad libitum*.

### Study Approval

All animal protocols were approved by the Institutional Animal Care and Use Committee in accordance with the Guide for Laboratory Animal Practice under the Association for the Assessment and Accreditation for Laboratory Animal Care International.

### Gestational Exposure to Sidestream Cigarette Smoke (SS)

Adult (3–4 months old) male and female mice (BALB/c) were separately acclimatized to SS or filtered air (FA) for 2 weeks before being paired for mating under the same exposure conditions. Briefly, mice were exposed to whole-body SS or FA for 6 h/days, 7 days/weeks (total particulate matter 1.52 ± 0.41 mg/m^3^) using Type 1,300 smoking machine (AMESA Electronics, Geneva, Switzerland) that generated two 70 cm^3^ puffs/min from 2R1 cigarettes as described previously (9, 10). The dose of SS was approximately equivalent to the amount of SS a pregnant woman would receive by sitting in a smoking bar for 3 h/days throughout the gestational period (10). After pregnancy was established, male mice were removed and the pregnant mice continued to receive SS or FA until the pups were born. Immediately after the birth of pups the exposures were stopped. On the postnatal day 7, some animals were sacrificed by an intraperitoneal injection of 0.2 ml Euthasol. Some adult F1 mice from FA and SS groups were mated to obtain the F2 progeny as described previously (10). Representative results are presented using animal from two different sets of SS-exposure. At least 15 animals per group were used; each analysis used 5 mice/group and the analysis was repeated twice. Specific details are given under figure legends.

### Human Placenta Samples

Placentas were collected at the University of New Mexico Hospital (UNMH), Albuquerque, NM according to protocol #17-064 approved by the University of New Mexico Medical Center Institutional Review Board and Human Research Protection Office in accordance with the NIH guidelines. All donors had agreed to donate the tissues. We were able to collect 10 placentas in a span of 7 months (by Dr. A. Sheybani and Dr. V. Exil, both from UNMH) representing three controls (mothers who did not smoke during the pregnancy), 4 CS-exposed (mothers who smoked throughout the pregnancy), two first-trimester quitters (mothers who stopped smoking during the first trimester of pregnancy), one false-control (mother who claimed to have quitted smoking during pregnancy, but the placenta had high level of cotinine). A 3 cm^3^ section of each placenta was dissected and frozen immediately for RNA and protein assays. Rest of the placentas were kept at −80°Cuntil use. Tissue slides (5 μm) were prepared by the institutional Pathology Core.

#### Determination of Cotinine Levels in Placentas

The smoking status of the mothers was confirmed by determining the cotinine levels in the placental tissues using the cotinine ELISA kit (Calbiotech Inc., CA) with a sensitivity of 5 ng/ml. Immunoblots were developed using placental homogenates.

#### Assays for H_2_S Synthesizing Enzymes

The expression of CSE, CBS, and 3MST was determined by WB analysis, IF-IHC, and/or qPCR. Assay details are given under relevant figure legends.

### Immunostaining and Immunofluorescent Imaging

For immunohistochemical (IHC) staining, deparaffinized and hydrated lung and placental tissue sections were washed in 0.05% v Brij-35 in PBS (pH 7.4) and immunostained for antigen expression as described previously (41). Briefly, the antigens were unmasked by steaming the sections in 10 mM Citrate buffer (pH 6.0) followed by incubation in a blocking solution containing 3% BSA, 1% Gelatin and 1% normal donkey serum with 0.1% Triton X-100 and 0.1% Saponin. Serial sections were stained with antibodies to Vimentin, E-cadherin, and ZO-1 (Invitrogen Inc., Carlsbad, CA), or isotype control antibodies. The immunolabelled tissues were detected using respective secondary antibodies conjugated with fluorescent dyes (Jackson ImmunoResearch Lab Inc., West Grove, PA). Where indicated, the sections were stained with 4′,6-diamidino-2-phenylindole (DAPI) containing Fluormount-G (SouthernBiotech, Birmingham, AL) to visualize nuclei. Immunofluorescent images were captured with BZX700 Microscopy system (Keyence, Tokyo, Japan). Specific details are given under appropriate figure legends.

### Western Blot Analysis

Western blot (WB) analysis of mouse lung and human placenta homogenates was carried out as described previously (10). Briefly, lung or placental tissues were homogenized in RIPA buffer and the protein content of the extracts was determined by the BCA Protein Assay Kit (Pierce, Rockford, IL). The homogenates were run on SDS-PAGE on 10% precast polyacrylamide gels (BioRad Lab, Hercules CA). The gels were transferred electrophoretically to nitrocellulose membranes (BioRad Lab). The blots were incubated with the respective antibodies. The mouse anti-β actin antibody (Santa Cruz Biotech) was used as the house-keeping protein. After incubating with an appropriate secondary antibody, the blots were developed with Amersham ECL Western Blotting Detection Reagent (GE Healthcare Bio-Science Corp. Piscataway, NJ) and the images were captured by Fujiform LAS-4000 luminescent image analyzer (FUJIFILM Corporation, Tokyo). Densitometry was used to quantitate the expression of specific proteins and expressed as the protein/β-actin band ratio.

### Quantitative Real-Time PCR (qPCR)

Total RNA was extracted by using a ToTALLY RNA kit (catalog number AM1910; Ambion, Austin, TX, USA). RNA samples were quantified by using a NanoDrop spectrophotometer and quality was analyzed on an RNA Nano-drop by using the Agilent 2100 bioanalyzer (Agilent Technologies). Synthesis of cDNA used 1 μg of total RNA in a 20 μl reaction mixture and TaqMan Reverse Transcription Reagents kit from ABI (catalog number N8080234; Applied Biosystems). qPCR amplification (performed in triplicate) used 1 μl of cDNA in a total volume of 25 μl of Faststart Universal SYBR green master mix (Roche Applied Science #04913850001). The mRNA sequences for CSE, and CBS for mouse and human reported under GenBank accession numbers NM145953 (CSE mouse), NM144855.3 (CBS mouse), NM_001902 (CSE human), and NM000071 (CBS (human) and were used to design primers for qRT-PCR assay (42–44). Expression of 3MST mRNA was performed using total RNA from lung and placental tissues by qPCR analysis and One-Step Real-Time PCR MasterMix containing TaqMan probes and a specific-labeled primer/probe set (Applied Biosystems). 18S RNA was used as housekeeping gene for normalization. PCR assays were run in the ABI Prism 7500 Sequence Detection System. Triplicate cycle threshold (*C*_T_) values were analyzed using the comparative *C*_T_ (ΔΔ*C*_T_) method as per manufacturer's instructions (Applied Biosystems). The amount of target (2^−Δ*ΔCT*^) was obtained by normalization to the endogenous reference (18S) sample. RNA isolation, primer design, and qRT-PCR assays were performed using the Molecular Genomic Core, UTMB, Galveston, TX.

### Statistical Analysis

Grouped results were expressed as mean ± SD and *p* ≤ 0.05 were considered significant. The data were normalized via natural log transformations and when the data was normally distributed, statistical significance among the groups was determined by one-way ANOVA with Bonferroni correction with multiple pairwise comparisons. When the data was not normally distributed, we used Kruskal-Wallis assessment on ranks followed by Dunn's multiple comparison tests. Student's *t*-test was employed for comparison between two groups at 95% confidence interval using Prizm software (GraphPad Software Inc., San Diego, CA). *p* ≤ 0.05 was considered statistically significant.

## Results and Discussion

### Gestational SS Inhibits EMT in the F1-Progeny Lung

**Figure 1:**
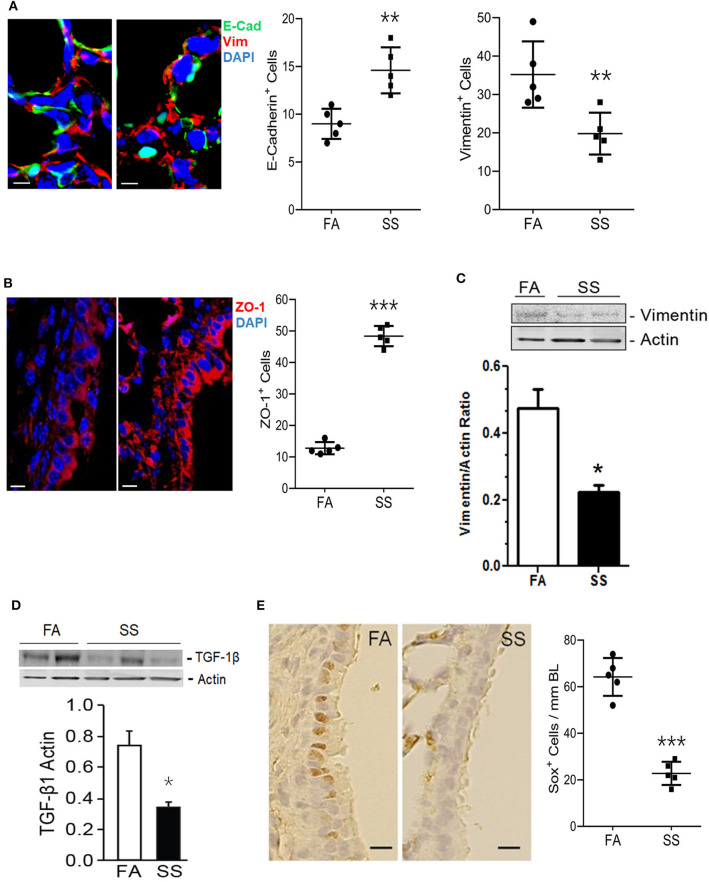
Gestational exposure to SS inhibits EMT in the mouse F1 lung. **(A)** Representative micrographs of lung sections from mice exposed gestationally to filtered-air (FA) or side-stream cigarette smoke (SS) and co-stained with vimentin (red) and cadherin (green); DAPI-stained nuclei (blue). E-Cadherin^+^ and Vimentin^+^ cells per unit area (18,000 μm^2^) were counted blind using NDP View on a Nanozoomer (Hamamatsu Photonics Inc.). **(B)** Representative micrographs of lung sections from gestationally FA or SS-exposed mice and stained with ZO-1 (red). ZO-1^+^ cells (12,400 μm^2^; NDP scanner). **(C)** Representative Western blot of lung tissue homogenates (70 μg) from FA or SS-exposed mice and probed with anti-vimentin antibody. Lower panel is the densitometry of the blot and expressed as Vimentin/Actin ratio. **(D)** Western blot analysis of lung tissue homogenates (70 μg) from FA or SS-exposed lungs probed with anti-TGF-β1 antibody (Cat# ab92486, Abcam). Lower panel is densitometry of the blot presented as TGF-β1/Actin ratio. **(E)** Representative image of the lung sections (5 μm) stained with anti-Sox2 antibody and detected by immunohistochemical staining. Right panel shows Sox^+^ cells (17,000 μm^2^; NDP scanner) counted blind. Data shown as mean±SD (*n* = 5/gp; *p < 0.05; **p < 0.01; ***p < 0.001).

Gestational exposure to SS impairs alveolarization and promotes BPD in the progeny, and these effects are transmitted to the F2 progeny and associated with suppressed levels of HIF-1α (11, 34). EMT is a biological process that allows epithelial cells to assume mesenchymal phenotype, which is critical for normal alveolarization (45) and regulated by HIF-1α, TGF-β, and VEGF (38, 39, 46, 47). VEGF promotes angiogenesis that stimulates EMT and alveolarization (48, 49) and intratracheal transplantation of mesenchymal stem cells attenuate lung injury in newborn mice (50). During embryogenesis and organ development, epithelial markers such as E-cadherin and ZO-1 are decreased and mesenchymal markers such as vimentin are increased (48, 51). The transcription factor HIF-1α promotes synthesis of TGF-β–the most potent inducer of EMT (52, 53) and HIF-1α is potently reduced in gestationally SS-exposed lungs (11). To determine whether gestational SS affected EMT, we determined the lung levels of epithelial (E-cadherin and ZO-1) and mesenchymal (vimentin) cell markers by IHC and Western blot analysis in 7-days old lungs from control and gestationally SS-exposed mice. Compared to control lungs, levels of E-cadherin (Figure 1A) and ZO-1 (Figure 1B) were significantly higher than those of vimentin (Figures 1A,C) in gestationally SS-exposed lungs. Moreover, the concentrations of TGF-β by Western blot analysis (Figure 1D) and of the anti-asthmatic factor SOX2 by IHC (Figure 1E), were significantly lower in gestationally SS-exposed lungs. SOX2 is a pluripotent transcription factor in bronchoalveolar progenitors, which promotes the Club cells to express Clara-cell secretory protein (CCSP) and surfactant protein-C (SP-C) (54). CCSP and SP-C are suppressed by gestational SS (10) and reduced SOX2 and CCSP levels are associated with higher risk of asthma (55); humans and mice deficient in CCSP, exhibit airway hyperresponsiveness (56). Together, these results suggest that gestational exposure to CS inhibits EMT and is associated with decreased numbers of SOX2-positive Clara cell progenitors.

### Gestational SS Suppresses CSE and CBS in the F1 and F2 Progeny Lungs

H_2_S is required for normal angiogenesis and alveolarization (16, 25) and produced in the periphery mainly by CSE and CBS (12). H_2_S attenuates lung injury (15, 57) and CSE deficiency exacerbates airway hyperreactivity (44) and impairs alveolarization. Impaired angiogenesis and alveolarization in CSE- and CBS-deficient mice are partially restored by H_2_S donor compounds (16). Moreover, H_2_S levels are lower in the exhaled air from asthma and COPD patients and correlates with lower FEV_1_ (58). Expression level of H_2_S enzymes is reported to be tissue specific. Thus, the brain and the vascular endothelium have a strong expression of CBS and CSE, respectively; however, both tissues also express 3MST (22). The situation in the lung is somewhat confusing. Lungs were reported to primarily express CSE (21); however, human lung cell lines such as A549 (26) and the lungs from cow and sea lions express all the three H_2_S synthesizing enzymes (24).

**Figure 2:**
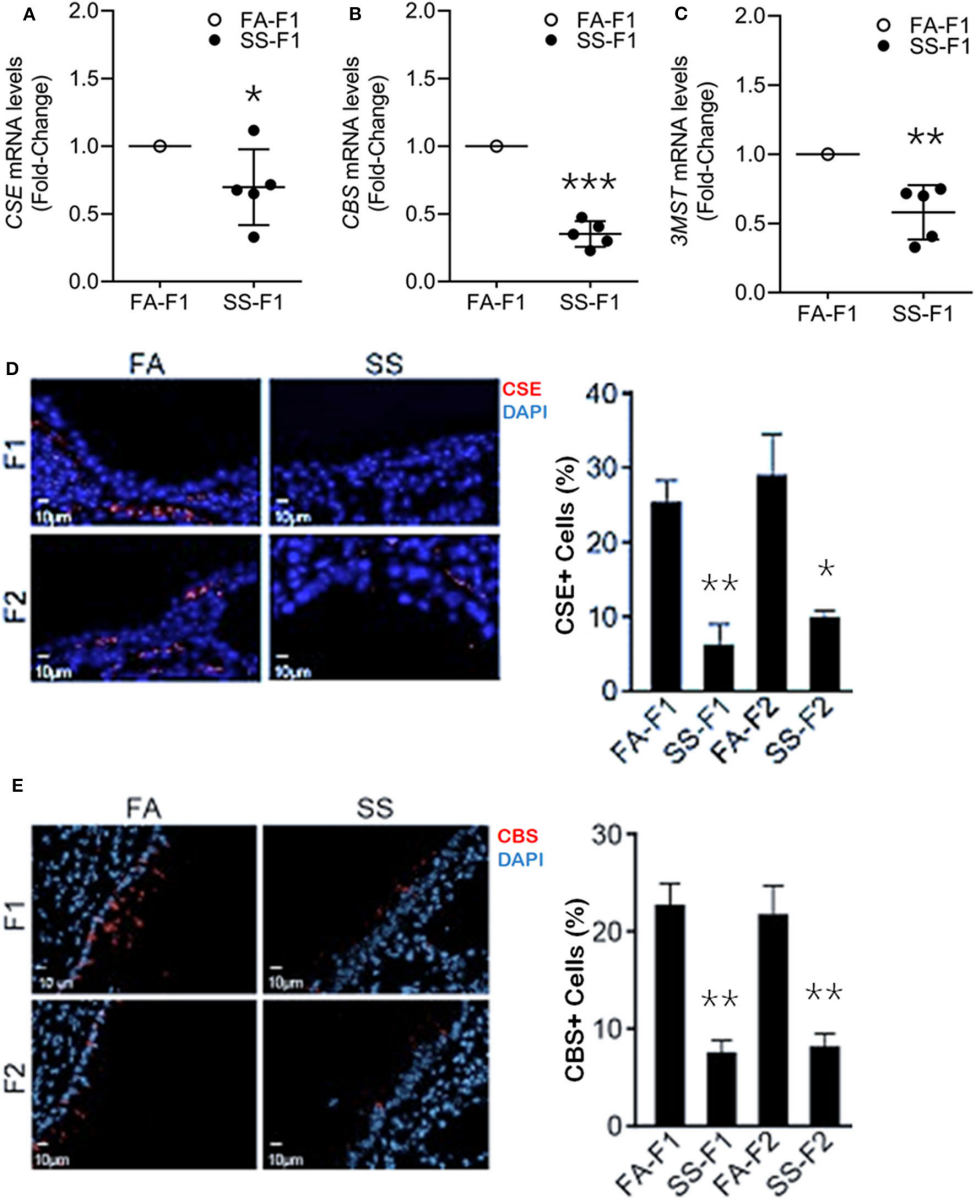
Gestational SS suppresses H2S biogenesis enzymes, CSE, CBS and 3MST in 7d old F1 mouse lung. Relative mRNA levels of *CSE* **(A)**, *CBS* **(B)**, and *3MST* **(C)** in F1 lungs exposed gestationally to FA or SS. **(D)** Representative micrographs of lung sections from F1 and F2 progenies exposed gestationally to SS or FA. Sections were stained for CSE (red) and DAPI (blue). Right pane is quantitation of CSE^+^ cells (%) in each group. **(E)** Micrographs of lung sections from F1 and F2 stained for CBS (red) and DAPI (blue). Right panel shows quantitation of CBS^+^ cells (%) in each group. Data shown as mean ± SD (*n* = 5/gp; *p < 0.05; **p < 0.01; ***p < 0.001).

**Figure 3:**
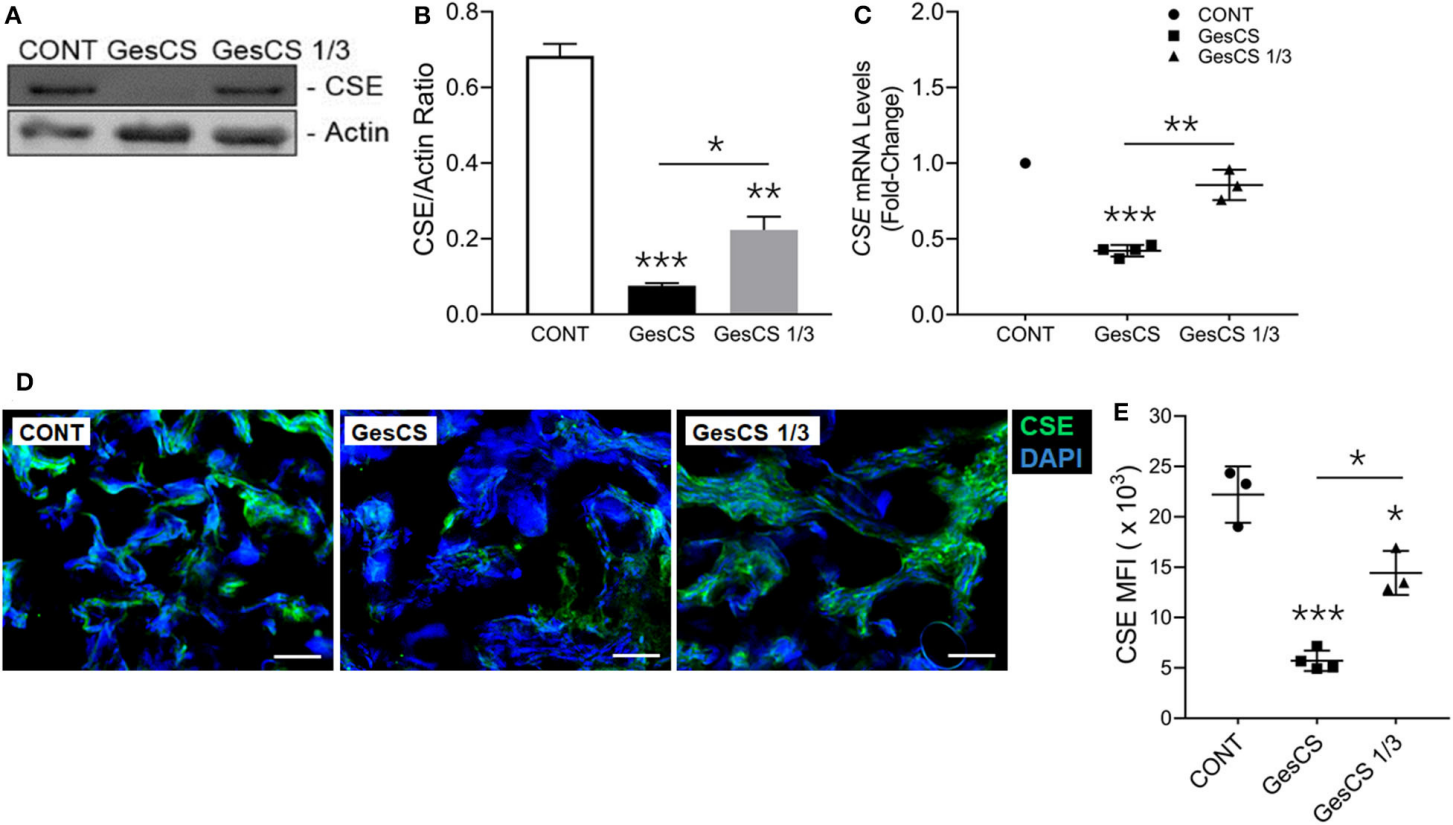
Gestational exposure to CS suppresses H2S biosynthetic enzyme CSE in human placenta. Placentas were analyzed for CSE expression using WB, qPCR and IF-IHC staining. **(A)** Western blot analysis of the placental tissue homogenate (150 μg protein) with anti-CSE antibody (Abcam, MA, USA). **(B)** Densitometry of CBS normalized to β-actin levels. **(C)** *CSE* mRNA detection by qPCR and expressed relative to CONT group. **(D)** Representative micrographs showing placental CSE (green) along with DAPI-stained nuclei (blue), scale−10 μ. **(E)** Quantification of CSE expression by MFIs (mean fluorescence intensity). CONT, control non-smoker; GesCS, cigarette smoking during whole pregnancy; GesCS1/3, CS exposure during first trimester; Data shown as mean ± SD (*n* = 3–4/gp; *p < 0.05; **p < 0.01; ***p < 0.001).

To ascertain whether gestational SS affected H_2_S production in the lung, we determined the mRNA levels of CSE (Figure 2A) and CBS (Figure 2B) and 3MST (Figure 2C) by qPCR in 7-day-old lungs from control and SS-exposed mice. Gestational CS inhibited mRNA levels of CSE, CBS, and 3MST, which would decrease the level of H2S in the lung and increase the risk of inflammatory lung diseases in these animals.

Gestational CS/SS/nicotine increases the risk of asthma and/or BPD transgenerationally in humans and animals (10, 59–61). To determine whether the transgenerational pro-asthmatic/pro-BDP effects of gestational SS were related to changes in H_2_S, 7-days old lungs from control and gestationally SS-exposed F1 and F2 mice were analyzed for CSE (Figure 2D) and CBS (Figure 2E) levels by IHC staining. Results showed that control lungs contained about equal numbers (~25%of total cells) of CSE- and CBS-positive cells, and gestational exposure to SS significantly reduced the number CSE/CBS-positive cells in both F1 and F2 animals. Thus, as reported for HIF-1α, angiogenesis, and alveolar volumes (11), gestational SS suppresses the levels of CSE and CBS in F1 progeny and the effects are transmitted to F2. Given the relationship between HIF-1α, TGF-β, EMT, angiogenesis, alveolarization, BPD, AA, and H_2_S, it is likely that the CS-induced proinflammatory lung responses in F1 and F2 progenies are related to changes in H_2_S levels regulated by H_2_S synthesizing enzymes. These data suggest that mouse lungs contain all three H_2_S synthesizing enzymes and gestational exposure to CS suppresses their expression. Reduced levels of these enzymes has the potential to promote lung diseases such as asthma and BPD.

### Placentas From Women Who Smoked During Pregnancy Express Low Levels of H_2_S Synthesizing Enzymes

Children from women who smoke during pregnancy have increased risk of AA and BPD (4–7) and herein our results suggest that the increased susceptibility may correlate with decreased levels of H_2_S synthesizing enzymes. Thus, the lung levels of H_2_S enzymes at birth may predict the risk of AA and BPD in children; however, it is unrealistic to obtain lung samples from newborn babies. Because, H_2_S synthesizing enzymes are present in most tissues (12, 62), we ascertained whether the enzymes were present in human placentas and, if so, whether smoking during pregnancy affected their expression. We were able to obtain 10 human placentas representing 3 controls (mothers who did not smoke during the pregnancy), four CS-exposed (mothers who smoked throughout the pregnancy), two first-trimester quitters (mothers who stopped smoking during the first trimester of pregnancy), one false-control (mother who claimed to have quitted smoking during pregnancy, but the placental showed high cotinine). Cotinine was determined on all placentas by ELISA to ensure that the tissues were from smoking/non-smoking mothers. CSE expression was determined by WB analysis, qPCR, and IF-IHC; CBS by WB and IF-IHC, and 3MST by qPCR analysis.

Immunoblot analysis of the placental homogenates from mothers who smoked throughout the pregnancy (GesCS) showed very low expression of CSE as compared to control non-smokers (CONT) or the mothers who quit during the 1st trimester (GesCS1/3) (Figures 3A,B). Similarly, as determined by qPCR analysis, CSE-specific mRNA content of GesCS placentas was significantly lower than CONT and GesCS1/3 (Figure 3C). Although the protein content of CSE in GesCS1/3 was higher than GesCS, it was still significantly lower than CONT (Figure 3B), suggesting that quitting smoking during the first trimester may be beneficial; however, the effects are not totally reversible and may persists after the birth. CSE expression was further confirmed by immunostaining of placental sections showing a 4-fold lower expression of CSE in GesCS than CONT; CSE expression in GesCS1/3 placentas was intermediate between CONT and GesCS (Figures 3D,E).

**Figure 4:**
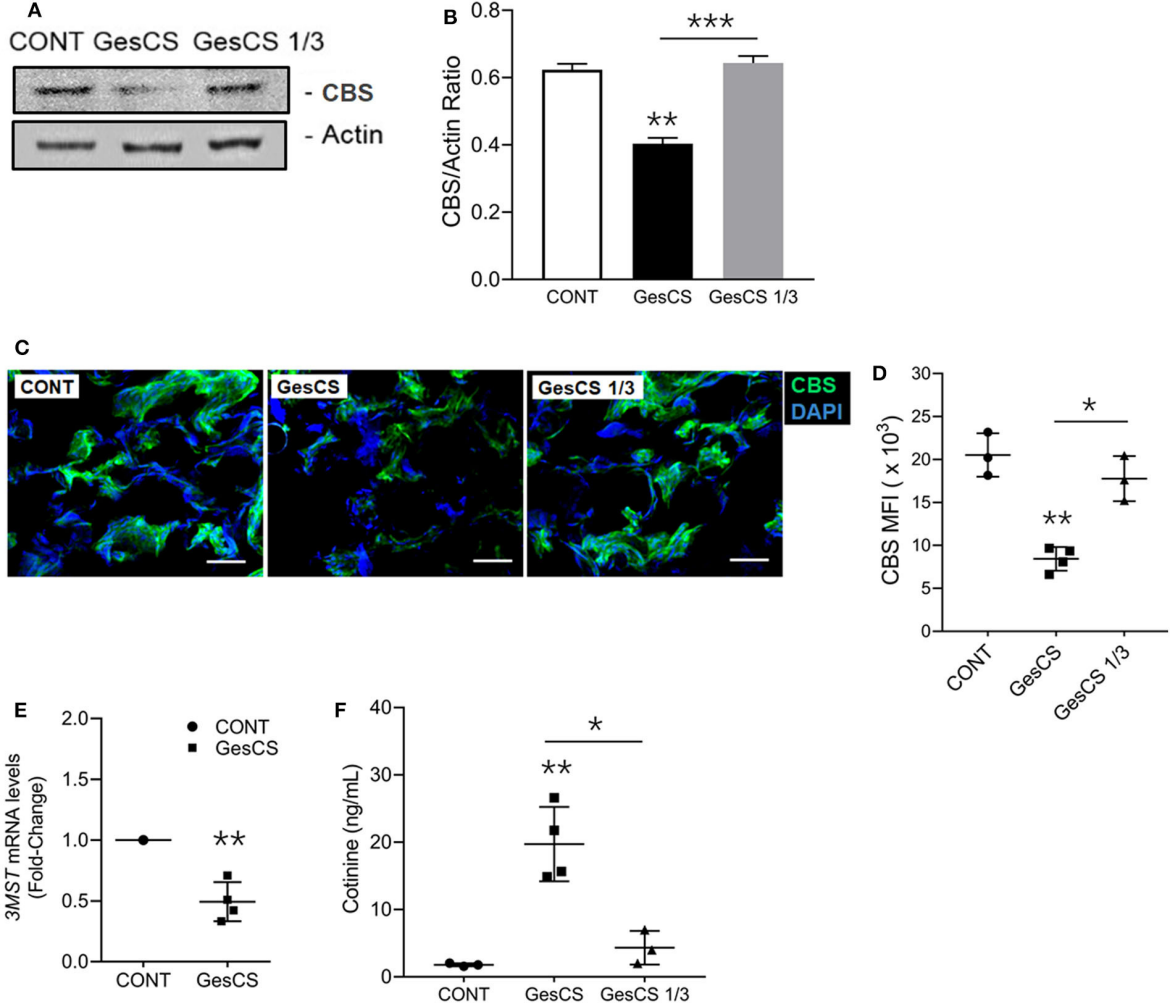
Gestational exposure to CS suppresses CBS and 3MST in human placenta. **(A)** Western blot analysis of placental tissue homogenates (150 μg protein) probed with anti-CBS antibody (Abcam, MA). **(B)** Densitometry of CBS normalized to β-actin. **(C)** Representative micrographs showing placental CBS (green) and DAPI-stained nuclei (blue), scale−10 μ **(D)** Quantification of CBS expression by MFI (mean fluorescence intensity) of CBS-immunoreactive fluorescence. **(E)** Quantitative RT-PCR of *3MST* mRNA expression *(n* = 5). **(F)** The smoking status of mother's was ascertained by the cotinine levels in the placental homogenate using cotinine ELISA kit (Calbiotech Inc., CA) with sensitivity of 5 ng/ml. CONT, non-smoker control; GesCS, cigarette smoker during pregnancy; GesCS1/3, CS exposure during first trimester; Data shown as mean ± SD (*n* = 3–4/gp; *p < 0.05; **p < 0.01; ***p < 0.001).

WB analysis also indicated that the expression of CBS was lower in GesCS than CONT or GesCS1/3 (Figures 4A,B). Furthermore, IHC analysis of CBS in placentas showed the expression was 3-fold lower in GesCS than CONT; however, the difference between CONT and GesCS 1/3 groups was not statistically significant (Figures 4C,D). We also examined the status of 3MST mRNA expression in human placentas by qPCR. Like CSE and CBS, exposure to cigarette smoke significantly inhibited the expression of 3MST (Figure 4E) indicating that, like CSE and CBS, gestational CS also downregulates 3MST expression in human placentas.

The smoking status of the mothers who donated the placentas was verified by measuring the cotinine levels (Figure 4F) and, in general, corroborated their assertion. However, we observed one outlier, where the WB and qPCR analyses of the placenta indicated very low levels of CSE (data not shown), yet the donor claimed to have quitted smoking during the pregnancy. The placenta contained high cotinine levels and was not included in the analyses. Sadly, it is not uncommon for the mothers to falsely assert quitting smoking during the pregnancy (3, 63).

**Figure 5:**
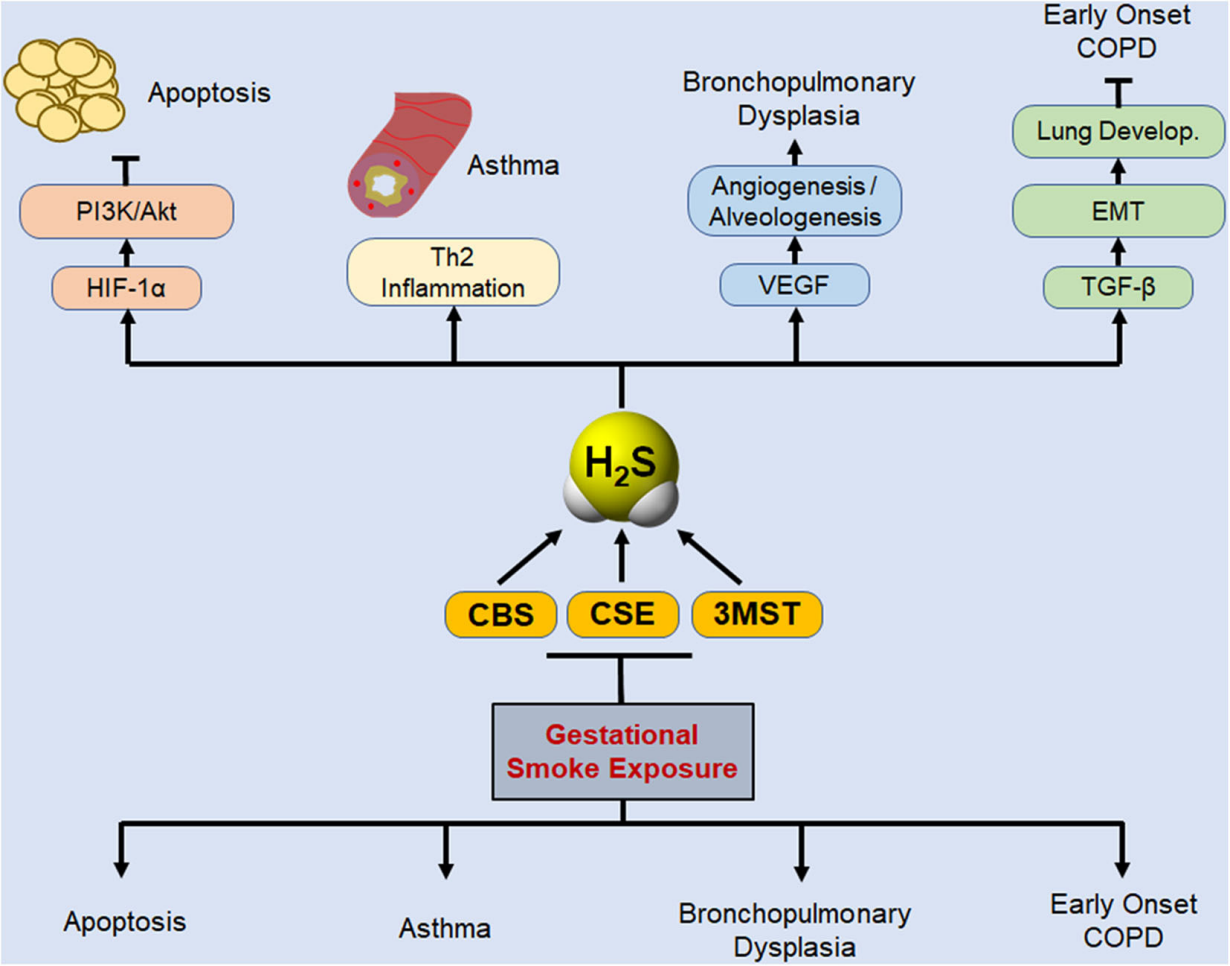
Schematic representation of potential protective role of biosynthesized H_2_S in basic lung pathophysiologies that are disrupted by gestational exposure to CS. Gestational exposure to CS is primarily linked to four major pulmonary predicaments: cell apoptosis, asthma, BPD, and susceptibility to early development of COPD. Lung cell apoptosis is associated with allergic inflammation (18) and decreased levels of HIF-1α and PI3K/Akt (34). PI3K/Akt inhibits apoptosis and promotes cell proliferation (67), and activation of Akt protects the neonatal lung against injuries (68). H2S donors inhibit apoptosis, attenuate lung damage, and promote normal lung development (21, 69). Asthma development as a consequence of gestational CS exposure or the deficiency of H_2_S enzymes is associated with increased Th2 inflammation (9, 17, 70), and exogenous H_2_S was shown to reverse the exacerbated asthma response in CSE-deficient mice (17). Gestation SS suppresses VEGF and angiogenesis, leading to impaired alveolarization and BPD (11, 34), and H_2_S stimulates VEGF expression and angiogenesis, (71), and alveolarization (72, 73). Maternal smoking affects lung development and has been linked to early onset of COPD in the progeny (74, 75). TGF-β is critical for EMT and normal lung development (31, 53) and herein we have shown that gestational CS downregulates TGF-β and inhibits EMT. Thus, gestational exposure to CS downregulates H_2_S synthesizing enzymes that in turn may increase the susceptibility of children to respiratory diseases associated with gestational exposure to CS.

The current study does not clearly define the stage(s) of pregnancy, where the fetus is completely resistant to the effects of CS on placental H_2_S enzymes. While the epidemiological evidence strongly suggests that CS exposure during pregnancy promotes wheeze and asthma in children (64), but the identity of the susceptible stage(s) of the pregnancy is not unequivocal and may vary from first trimester (6) to third trimester (5). Our data with placental levels of H_2_S enzymes suggest that the effects of smoking during first trimester are moderate, but not negligible; however, we have not correlated these levels to the actual incidence of asthma in the progeny. Interestingly, perinatal exposure to nicotine induces asthma in rats (61), suggesting that late stages of embryonic development might be more sensitive to gestational CS. Nonetheless, it is highly likely that there is a correlation between placental levels of H_2_S enzymes and the risk of asthma/BPD in children and H_2_S or H_2_S-donor compounds may have therapeutic value to reduce this risk. The manner by which H_2_S inhibits allergic asthma is related to its ability to suppress Th2 immune responses (65) and CSE deficient mice have elevated GATA3 nuclear content, higher levels of Th2 cytokines, and exaggerated asthma response; H_2_S donors attenuate asthma (17, 66). Thus, gestational exposure to CS downregulates H_2_S synthesizing enzymes that in turn may increase the susceptibility of children to respiratory diseases associated with gestational exposure to CS. Taken together, the data presented herein provide a basic outline of the potential interaction between gestational CS exposure, *de novo* synthesis of H_2_S, and development of lung developmental diseases as described schematically in Figure 5.

## Research Impact

Exposure to cigarette smoke (CS) during pregnancy impairs epithelial-mesenchymal transition (EMT) and angiogenesis in the lung, increasing the risk of allergic asthma and bronchopulmonary dysplasia (BPD), transgenerationally. Hydrogen sulfide (H2S), a recently recognized gasotransmitter, promotes angiogenesis and inhibits asthma and alveolar simplification. H_2_S is synthesized by cystathionine-β-synthase (CBS), cystathionine-γ-lyase (CSE), and 3-mercaptopyruvate sulfur transferase (3MST). Results presented herein show that exposure of mice to CS during pregnancy suppressed the lung expression of CSE, CBS, 3-MST, and the CS-induced suppression of CSE and CBS was transmitted to F2. Similarly, smoking during pregnancy downregulated the expression of CSE, CBS, and 3MST in human placentas; the downregulated expression of the enzymes might be a biomarker for asthma susceptibility in children.

## Data Availability Statement

All datasets generated for this study are included in the article.

## Ethics Statement

The studies involving human participants were reviewed and approved by University of New Mexico Medical Center's Institutional Review Board and Human Research Protection Office in accordance with the NIH guidelines. The patients/participants provided their written informed consent to participate in this study. The animal study was reviewed and approved by Lovelace Respiratory Research Institute IACUC.

## Author Contributions

SS performed the experiments, analyzed the data, and wrote the manuscript, DD, MM, AS, and TI analyzed the data, VE, HA, and VR performed the sample analysis and analyzed the data. RG analyzed the data and wrote the manuscript. HC performed the sample analysis, analyzed the data, and wrote the manuscript. MS designed the studies, analyzed the data, and wrote the manuscript. All authors reviewed the manuscript.

## Conflict of Interest

The authors declare that the research was conducted in the absence of any commercial or financial relationships that could be construed as a potential conflict of interest.

## Abbreviations

AA: allergic asthma
BPD: bronchopulmonary dysplasia
CBS: cystathionine-β-synthase
CCSP: Clara-cell secretory protein
COPD: chronic obstructive pulmonary disease
CS: cigarette smoke
CSE: cystathionine-γ-lyase
CS: cigarette smoke
EMT: epithelial mesenchymal transition
FA: filtered air
GesCS: gestational cigarette smoke
GesCS1/3: mothers who quit smoking during the 1^st^ trimester
HIF-1α: hypoxia Inducible factor-1α
H_2_S: Hydrogen sulfide
3MST: 3-mercaptopyruvate sulfur transferase
MFI: mean fluorescence intensity
NaHS: sodium hydrosulfide
SS: secondhand CS
SP-C: surfactant protein-C
TGF-β: transforming growth factor beta
ZO-1: zonula occludens-1.
